# Patterns of women’s work resumption and economic independence after childbirth: A longitudinal study

**DOI:** 10.1177/14034948251394645

**Published:** 2025-12-02

**Authors:** Lindsey Van Der Meer, Kajal S.C. Mohabier, Adja J.M. Waelput, Alex Burdorf, Eric A.P. Steegers, Loes C.M. Bertens

**Affiliations:** 1 Department of Obstetrics and Gynaecology, Erasmus University Medical Centre Rotterdam, The Netherlands; 2 Department of Public Health, Erasmus University Medical Centre Rotterdam, The Netherlands; 3 Division of Obstetrics and Prenatal Medicine, Department of Obstetrics and Gynaecology, Rotterdam, Erasmus University Medical Centre Rotterdam, The Netherlands

**Keywords:** Pregnancy, childbirth, economic independence, sick leave, employment

## Abstract

*Background:* Women often reduce their working hours after childbirth, or, in some cases, exit the workforce altogether. This adjustment affects their immediate economic independence, with implications for their career trajectory and long-term financial stability. We aim to study the patterns of women’s work resumption in the first year after childbirth, including the role of sick leave during pregnancy. We further aim to identify the key factors contributing to women’s economic independence one year after childbirth. *Methods:* Drawing on data from Statistics Netherlands and the Dutch Perinatal Registry, we used descriptive analysis to examine the working hour patterns from onset of pregnancy to one year after childbirth. Additionally, multinomial logistic regression assessed how individual and work-related factors related to having employment with economic independence, having employment without economic independence, or having exited the workforce one year after childbirth. *Results:* Data on 114,722 Dutch employed women who delivered in 2016 showed a notable shift in employment patterns one year after birth: over half reduced their working hours, with 12.2% leaving the workforce entirely. These patterns were particularly pronounced among women with sick leave during pregnancy. Furthermore, factors such as lower education, fewer working hours, and not cohabiting with a partner were associated with being employed but not economically independent after childbirth. ***Conclusions:*** **Pregnancy and delivery have a durable impact on women’s workforce participation, which affects their economic independence. Strategic workplace and family policies might support retaining employment after childbirth and mitigate adverse economic effects, particularly for those with sick leave during pregnancy.**

## Background

Employment is a fundamental right for individuals and has numerous advantages, including economic independence, defined as the ability to be financially self-sufficient (see Supplementary material File 1 online) [1,2]. Despite the benefits of employment, pregnant women in the workforce often face unique challenges, including pregnancy discrimination and balancing their professional responsibilities alongside the physical and hormonal changes [3,4]. These challenges are exacerbated by a persistent unequal distribution of caregiving responsibilities between men and women, ultimately intensifying existing gender inequalities in labour market participation and career progression [5,6].

In the Netherlands, a range of policies, as outlined in Supplementary File 1, can provide women (financial) support to adapt to the altered circumstances that accompany pregnancy and childbirth. Regarding maternity leave, women in paid employment are generally entitled to 16 weeks of leave with full income compensation. Since 2019, the leave for employed partners has increased from two days to one working week of fully paid leave, which can be supplemented with up to five additional weeks at 70% of the salary [7].

While these policies are intended to be supportive, they may paradoxically disrupt women’s continuous workforce participation and economic independence by enabling prolonged leave, which poses subsequent challenges upon re-entry into the workforce [5]. Many women make career adjustments after childbirth to manage the new caregiving responsibilities. Factors such as age at first childbirth, educational attainment, family circumstances, partners’ employment status, and job prospects are commonly described in the literature as influencing these decisions [6,8]. Yet, it is less well known how these factors affect their economic independence and which factors contribute most to this outcome. Additionally, pregnancy complications, which are the most common cause of sick leave during pregnancy, can have lasting health effects [9]. These complications are known to increase the risk of cardiovascular and metabolic diseases later in life, which can, in turn, impact their professional life [9–11]. Although such complications can influence the resumption of employment after pregnancy, the impact of a broader range of sicknesses during pregnancy, whether pregnancy related or not, on employment resumption is unknown.

This study aims to explore the detailed patterns of women’s employment resumption in the first year after childbirth, with a particular focus on the role of sick leave during pregnancy. Moreover, it seeks to identify key factors that influence women’s economic independence in the first year after childbirth. The findings of this study can guide the development of policies to accommodate the convergence of parenthood and career development.

## Methods

### Data sources

The data for this study were derived from Statistics Netherlands (CBS) and the Dutch Perinatal Registry (Perined). CBS collates and disseminates statistical data on a range of societal issues by collecting data directly from individuals and businesses through a diverse set of methods [12]. Perined systematically collects detailed data on the perinatal process from midwives, gynaecologists and paediatricians, and aims to improve the quality of perinatal care by improving the understanding of care procedures and their outcomes [13]. For this study, we linked pseudonymised individual-level data for all persons residing in the Netherlands as documented in the Personal Records Database (BRP) across various registers maintained by CBS in their System of social statistical datasets [14]. CBS retrieves information on employment earnings, employment characteristics and social benefits from the Employee Insurance Agency, largely based on registers with individual monthly tax information. Data on personal characteristics and residential information are obtained from the BRP, while pregnancy outcome data were retrieved from the Perined registry. All analyses were conducted within CBS’s secure research environment. A formal approval from the ethics committee was not required.

### Subjects

Our study population consisted of women with a singleton viable pregnancy in 2016, defined as a birthweight above 500 g or a birth after 22+0 weeks of gestation, and who were registered in the BRP in the period from 2016 to 2019. In cases where a woman gave birth twice in 2016, only the first pregnancy was included. Additionally, only women with paid employment at the onset of pregnancy, and of whom monthly income data were available, were included in the analyses.

We restricted our study population to women with singleton pregnancies, as women with multiple pregnancies have a higher risk of medical complications, which may subsequently influence women’s employment trajectories after birth [15]. Furthermore, the 2016 birth cohort was chosen to allow a follow-up of three years to 2019, ensuring that the study period was not influenced by the profound labour market disruptions caused by the COVID-19 pandemic [16,17].

### Variables

#### Outcome variables

In the descriptive analysis, the outcome variable is employment status after childbirth, determined by whether a woman receives earnings from employment in a given month. For the multivariate multinomial logistic regression, the outcome variable centres on economic independence and is categorized into three groups: employment with economic independence, employment without economic independence, and exited the workforce. In the Netherlands, an individual is deemed economically independent if the personal income from employment meets or exceeds the statutory subsistence minimum (see Supplementary File 1), which equates to at least 70% of the legal minimum wage [18].

#### Descriptive and predictive variables

##### Baseline descriptives

To describe the population at baseline, we included the following individual characteristics:

- Maternal age (continuous: in years);
- Generational migration status (categorical: Dutch; first-generation; second-generation);
- Educational attainment (based on highest completed education, classified using the CBS Standard Educational Classification (SOI 2021) [19]; categorical: low; middle; high; unknown);
- Main source of income (categorical: paid employment; self-employed; benefits; other; no income/unknown);
- Cohabiting partner (categorical: yes; no);
- Parity (categorical: primiparous; multiparous);
- Duration of pregnancy at childbirth (calculated from the first day of the last menstrual period, continuous: in weeks);
- Perinatal mortality (from 22 weeks of gestation to seven days after birth; categorical: yes; no);
- Small for gestational age ((SGA) birthweight lower than the 10th percentile for gestational age; categorical: yes; no);
- Preterm birth (birth before 37 weeks of gestation; categorical: yes; no);
- Neighbourhood socioeconomic status ((SES) continuous).

The neighbourhood SES score is calculated by CBS based on various socioeconomic factors such as income (average income of residents and proportion of residents receiving social welfare benefits), employment rates (proportion of the employed working-age population), education levels (percentage of residents with different levels of educational attainment) and housing conditions (quality, ownership and affordability of housing) [20]. We did not have information regarding the mode of delivery as it was not available in the used registries.

##### Employment descriptives

To capture employment patterns and changes, work-related characteristics were included at two time points: at the onset of pregnancy, determined by the date of birth minus the pregnancy duration, and one year after childbirth. This allowed us to assess both baseline conditions and subsequent changes over time in relation to women’s economic independence. The characteristics included:

- Working hours per week (categorical: less than 12; 12–20; 20–25; 25–30; 30–35; 35 or more; not employed);
- Employment arrangement (categorical: fixed hours; flexible hours);
- Contract type (categorical: permanent; temporary; unknown);
- Sick leave (one or more consecutive days of sickness absence, categorical: yes; no);
- (Additional) Unemployment benefits (categorical: yes; no);
- (Additional) Social assistance benefits (categorical: yes; no);
- Working hours of cohabiting partner (categorical: less than 12; 12–20; 20–25; 25–30; 30–35; 35 or more; not employed);
- Economic independence (categorical: yes; no).

Furthermore, during pregnancy the following variables were considered:

- Sick leave (categorical: yes; no);
- Number of sick leaves (categorical: 1; 2; 3(+)).

##### Multinomial regression

For the multinomial regression analyses, the following characteristics were included as predictors:

- Individual characteristics: maternal age, generational migration status, educational attainment, parity, neighbourhood SES, cohabiting partner (measured at onset of pregnancy and one year after childbirth), SGA, preterm birth, and perinatal mortality.
- Work-related characteristics (measured at onset of pregnancy and one year after childbirth): working hours per week, employment arrangement, contract type, sick leave during pregnancy, and receipt of unemployment and social assistance benefits.

### Statistical analysis

Population characteristics were described using mean and standard deviation or number and percentages, as appropriate. These characteristics were stratified by two distinct stages: the onset of pregnancy and one year after childbirth. Patterns of leaving and re-entry into the workforce were analysed, tabulated and visualized for women in the study. Subsequently, these data were stratified based on sick leave during pregnancy.

Multivariate multinomial logistic regression was used to evaluate the predictors associated with achieving economic independence one year after childbirth among women who gave birth in 2016. Only data from women with no missing values for any of the predictors were used. Missing values were present in two variables: SGA (0.3%) and neighbourhood SES (0.6%). To ensure the model’s performance, the data were split into two subsets: the first subset containing 70% of the data to train the model, and the second subset containing 30% of the data to test the model. First, univariate logistic regression models evaluated the influence of each predictor separately. Next, multivariate logistic regression evaluated the combined influence of the predictor variables. The Variance Inflation Factor assessed multicollinearity among the predictors, with values above 5 considered as indicative for multicollinearity issues [21]. Accuracy of the model was depicted using a confusion matrix, a tabular representation that contrasts the actual versus predicted outcomes, summarizing true positives, true negatives, false positives and false negatives. Model accuracy values above 0.80 are typically considered as indicating strong classification performance [22]. Area under the curve evaluated the discriminative ability of the model to accurately predict economic independence one year after birth, with values above 0.70 generally regarded as acceptable. Model residuals were also inspected and various pseudo *R*^2^ metrics (Nagelkerke, McFadden) quantified the explained variance by our model and its predictive power [23]. In logistic regression, values above approximately 0.40 (Nagelkerke) and 0.20 (McFadden) are often considered to indicate acceptable to good model performance.

## Results

### Population description

A total of 161,656 women gave birth in 2016 (Table I). While the primary source of income for most of them was paid employment, a substantial portion (*n* = 50,605, 31.3%) had other main sources of income. Specifically, the majority of this group were either self-employed (*n* = 10,992, 6.8%) or reliant on some form of benefit (*n* = 16,524, 10.2%). An additional 23,089 (14.3%) women had either a different source of income or no income at all.

**Table I.** Baseline characteristics of women in paid employment at the onset of their pregnancy.

Baseline characteristics	Total group of women giving birth in 2016 (*N* = 161,656)	Women in paid employment at the onset of pregnancy (*n* = 114,722)
Age, mean (SD)	30.7 (4.7)	30.8 (4.4)
Generational migration status, *n* (%)		
Dutch	111,815 (69.2%)	89,393 (77.9%)
First-generation migrant	30,326 (18.8%)	12,679 (11.1%)
Second-generation migrant	19,515 (12.1%)	11,650 (11.0%)
Educational attainment, *n* (%)		
High	66,095 (40.9%)	55,789 (48.6%)
Middle	57,134 (35.3%)	41,704 (36.4%)
Low	19,326 (12.0%)	7452 (6.5%)
Unknown	19,101 (11.8%)	9772 (8.5%)
Main source of income, *n* (%)		
Paid employment	111,051 (68.7%)	110,360 (96.2%)
Self-employed	10,992 (6.8%)	1055 (0.9%)
Benefits	16,524 (10.2%)	1253 (1.1%)
Other	4086 (2.5%)	2054 (1.8%)
No income or unknown	19,003 (11.8%)	N/A
Cohabiting partner, *n* (%)		
Yes	135,142 (83.6%)	101,250 (88.3%)
Duration of pregnancy in weeks at childbirth, mean (SD)	39.1 (2.0)	39.1 (2.0)
Parity, *n* (%)		
Primiparous	71,452 (44.2%)	55,917 (48.7%)
Multiparous	90,204 (55.8%)	58,802 (51.3%)
Perinatal mortality, *n* (%)		
Yes	1013 (0.6%)	652 (0.6%)
SGA, *n* (%)		
Yes	13,252 (8.2%)	8351 (7.3%)
* Missing*	501 (0.3%)	344 (0.3%)
Preterm birth, *n* (%)		
Yes	8968 (5.6%)	6236 (5.4%)
Economic independence^a^, *n* (%)		
Yes	N/A	101,377 (88.4%)
Neighbourhood SES^b^		
Mean (SD)	–0.01 (0.2)	0.0 (0.2)
* Missing*	3632 (2.3%)	733 (0.6%)

a The threshold value for economic independence in 2016 amounted to €928.29 per month. Please note that economic independence was calculated only for the group of women who held paid employment.

b In 0.64% of the cases the neighbourhood SES was unknown. The SES-score is centred to zero, with a minimum score of –1.6 and a maximum score of 0.7, with a higher score representing a higher neighbourhood SES.

SGA: small for gestational age; N/A = not applicable; SES: socioeconomic status

Our study population consisted of the 114,722 women with paid employment. Their mean age was 30.8 years and nearly 80.0% were of Dutch origin. Regarding educational attainment, 48.6% achieved a high-level education, 36.4% a middle-level, 6.5% a low-level education, and for 8.5% the educational attainment was unknown. Approximately half of the women were primiparous and the mean gestational age at childbirth was 39.1 weeks. Incidences of adverse pregnancy outcomes, such as perinatal mortality, SGA and preterm birth, were 0.6%, 7.3% and 5.4%, respectively. The vast majority of the women were classified as economically independent at onset of pregnancy (88.2%).

### Work characteristics

In Table II, key data about the employment situation of women in our study sample at onset of pregnancy and one year after childbirth are presented. Initially, the majority of women (69.2%) worked part-time (< 35 h per week). Approximately 11.0% of women took sick leave during their pregnancy, with the majority (81.7%) experiencing a single period of sick leave. However, 17.0% had two instances of sick leave, and a smaller proportion (1.4%) had three or more instances. Notably, the proportion of women reporting sick leave increased sixfold one year after childbirth. The proportion of women in full-time employment (>35 h per week) dropped from 30.8% at the start of pregnancy to 11.3% one year after childbirth, thereby increasing the overall proportion of women working part-time to 76.5% one year after childbirth. This increase was most substantial in the 20–25 h per week category, which grew from 19.0% to 27.5%. Additionally, 12.2% of all employed women at the start of pregnancy had exited the workforce completely. These changes in working hours did not reverse in the subsequent two years (see Supplementary File 2). Furthermore, one year after childbirth, women more often complemented paid employment with additional benefits such as unemployment and social security benefits.

**Table II.** Work-related characteristics of women in paid employment (*N* = 114,722) at onset of pregnancy and one year after childbirth.

Characteristics	Onset of pregnancy	One year after childbirth
Working hours per week, *n* (%)		
<12 h	6924 (6.0%)	5552 (4.8%)
12–<20 h	11,846 (10.3%)	13,371 (11.7%)
20–<25 h	21,764 (19.0%)	31,529 (27.5%)
25–<30 h	16,687 (14.6%)	19,040 (16.6%)
30–<35 h	22,228 (19.4%)	18,279 (15.9%)
35 h or more	35,273 (30.8%)	12,968 (11.3%)
Not employed	N/A	13,983 (12.2%)
Employment arrangement, *n* (%)		
Fixed hours	103,309 (90.1%)	72,927 (63.6%)
Flexible hours	11,413 (10.0%)	27,812 (24.2%)
Not employed	–	13,983 (12.2%)
Contract type, *n* (%)		
Permanent	73,218 (63.8%)	74,825 (65.2%)
Temporary	41,463 (36.1%)	25,730 (22.4%)
Unknown	41 (0.0%)	184 (0.2%)
Not employed	N/A	13,983 (12.2%)
Sick leave, *n* (%)		
Yes	105 (0.1%)	676 (0.6%)
(Additional) Unemployment benefits, *n* (%)		
Yes	1906 (1.7%)	5941 (5.2%)
(Additional) Social assistance benefits, *n* (%)		
Yes	676 (0.6%)	1160 (1.0%)
Cohabiting partner, *n* (%)		
Yes	101,250 (88.3%)	106,921 (93.2%)
Working hours partner^a^, *n* (%)		
<12 h	1000 (1.0%)	886 (0.8%)
12–<20 h	1177 (1.2%)	1083 (1.0%)
20–<25 h	1548 (1.5%)	1713 (1.6%)
25–<30 h	2312 (2.3%)	2734 (2.6%)
30–<35 h	7678 (7.6%)	11,715 (11.0%)
35+ h	72,236 (71.3%)	72,114 (67.5%)
Not employed	15,299 (15.1%)	16,676 (15.6%)
Economic independence, *n* (%)		
Yes	101,230 (88.2%)	89,987 (78.4%)
No	13,492 (11.8%)	10,752 (9.4%)
Not employed	N/A	13,983 (12.2%)
Characteristics	During pregnancy	
Sick leave, *n* (%)		
Yes	12,448 (10.9%)	
Number of sick leaves, *n* (%)		
1	10,164 (81.7%)	
2	2111 (17.0%)	
3(+)	173 (1.4%)	

a These figures represent only the weekly working hours of partners of women who were cohabiting.

N/A: not applicable

### Employment patterns

Figures 1 and 2 illustrate the detailed employment patterns from the onset of pregnancy to one year after childbirth. When comparing the group of women having no sick leave during pregnancy with those having sick leave, distinct patterns emerge. Women who took sick leave were more likely to reduce their working hours or exit the workforce entirely. Among those who worked full-time (35 h or more) at the onset of pregnancy, 3.5% of women with sick leave continued working full-time one year after childbirth, compared with 6.8% of those without sick leave. Additionally, 39.5% of women who took sick leave exited the workforce completely, in contrast to the 6.8% among women who did not take sick leave.

**Figure 1.**
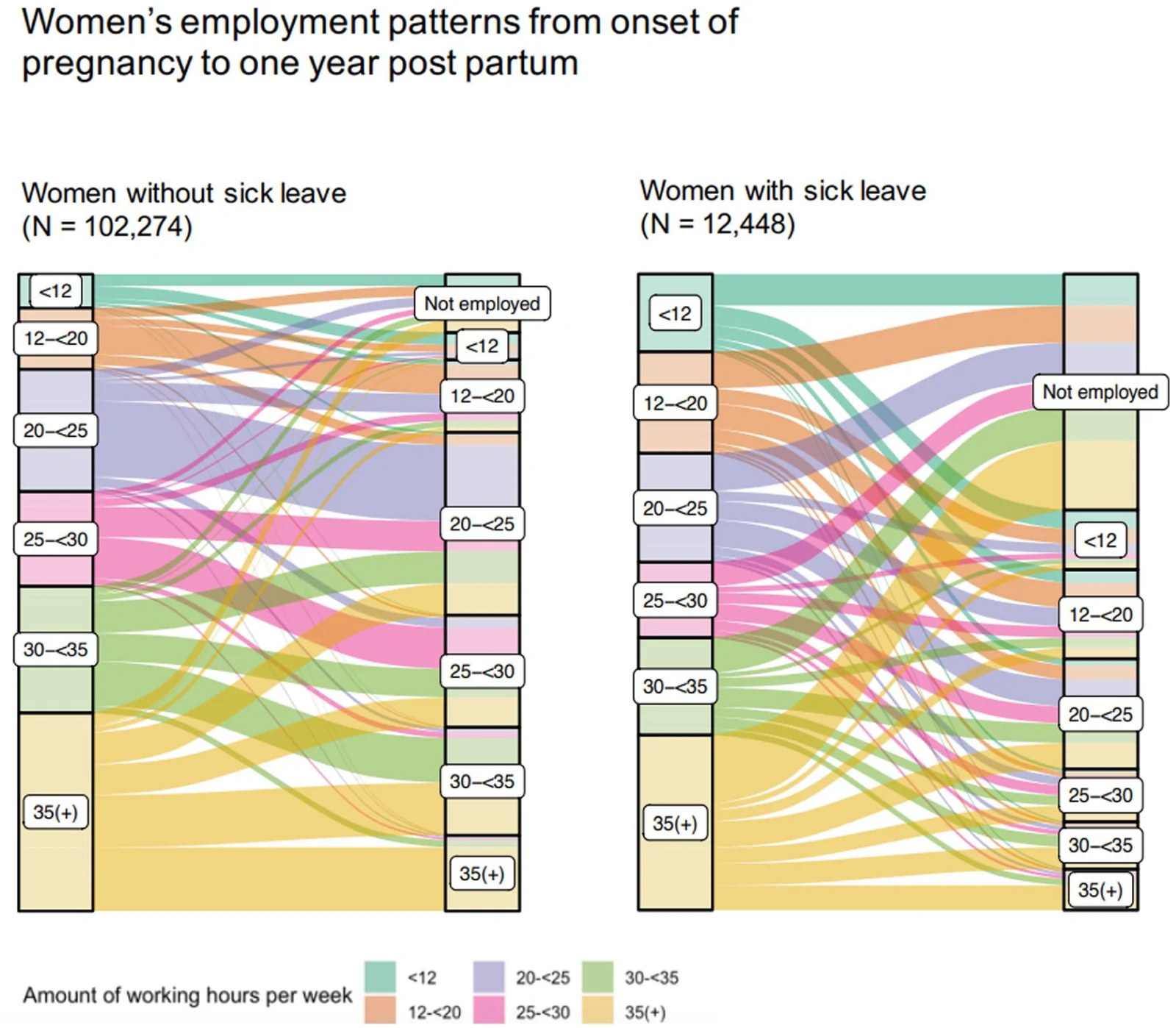
Flow diagram visualizing employment patterns of women in paid employment, from onset of pregnancy to one year after childbirth.

**Figure 2.**
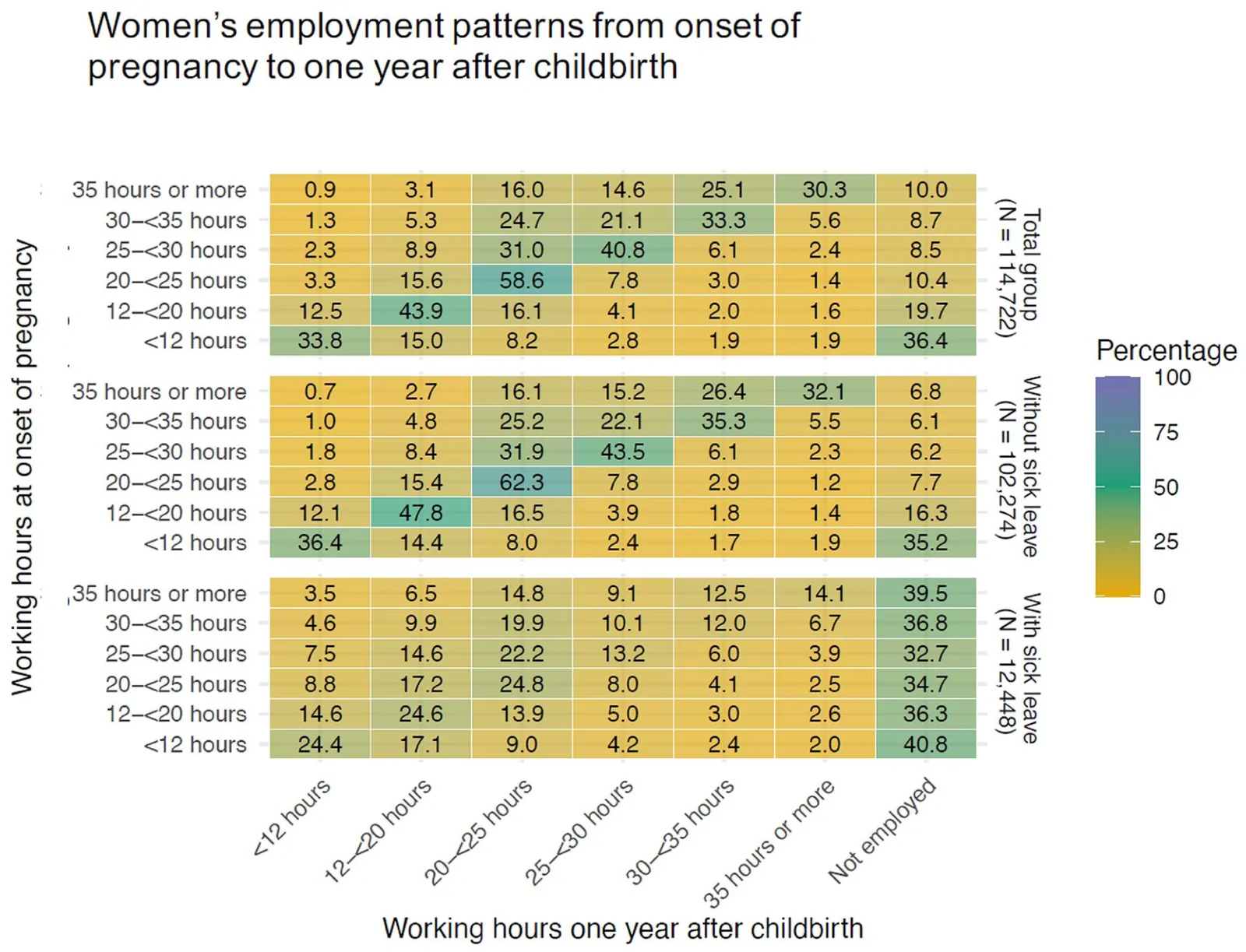
Matrix displaying employment patterns (%) of women in paid employment from onset of pregnancy to one year after childbirth.

Overall, more than half (51.7%) of the women reduced their working hours or exited the workforce. The most pronounced shift of reducing working hours was in the group that worked full-time at the beginning of their pregnancy. About 60.0% of this group reduced their working hours, mostly transitioning to working between 30 and 35 h. A little over 30.0% remained working full-time and 10.0% of them left the workforce. The majority of women who were working 20–25 h per week sustained their working hours after childbirth (58.6%). For women who took sick leave, there was a significant shift toward part-time work, particularly in the 12–20 h (24.4%) and 20–25 h (17.2%) categories. Notably, the largest share of women leaving the workforce are those who work a maximum of 12 h per week (nearly 37.0%).

### Economic independence

Table III shows the results of the multinomial logistic regression analyses. Univariate results are presented in Supplementary File 2; all variables were significantly associated with economic independence in the univariate models, except for perinatal mortality. In the multivariate analyses employed and economically independent women served as a reference group.

**Table III.** Univariate and multivariate multinomial logistic regression of economic independence one year after childbirth (*N* = 113,134).

Variables	Multivariate multinomial logistic regression	
	Employed without economic independence OR (95% CI)	Exited the workforce OR (95% CI)
Individual characteristics		
**Age**	0.92 (0.91–0.93)***	1.01 (0.51–1.97)
**Generational migration status**		
Dutch	Ref.	Ref.
First-generation migrant	1.54 (1.35–1.77)***	42.51 (42.36–42.66)***
Second-generation migrant	1.09 (0.95–1.24)	3.84 (3.83–3.84)***
**Educational attainment**		
Low	12.40 (10.77–14.29)***	0.06 (0.06–0.06)***
Middle	4.08 (3.70–4.49)***	0.46 (0.45–0.46)***
High	Ref.	Ref.
Unknown	5.23 (4.60–6.07)***	0.08 (0.08–0.08)***
**Cohabiting partner at onset of pregnancy**		
Yes	Ref.	Ref.
No	1.35 (1.18–1.55)***	0.04 (0.04–0.04)***
**Parity**		
Primiparous	Ref.	Ref.
Multiparous	0.83 (0.76–0.91)***	4.58 (4.51–4.64)***
**Neighbourhood SES**		
	0.75 (0.62–0.91)***	0.00 (0.00–0.00)^b^,***
**Perinatal mortality**		
Yes	–	–
No	–	–
**SGA**		
Yes	1.17 (1.02–1.35)*	0.00 (0.00–0.00)^b^***
No	Ref.	Ref.
**Preterm birth**		
Yes	1.18 (1.00–1.40)*	4.71 (4.71–4.71)***
No	Ref.	Ref.
**Cohabiting partner one year after childbirth**		
Yes	Ref.	Ref.
No	1.22 (1.02–1.45)*	0.02 (0.02–0.02)***
Work-related characteristics		
**Working hours per week at the onset of pregnancy**		
<12 h	2.71 (2.30–32.0)***	22.63 (22.55–22.70)***
12–<20 h	1.70 (1.48–1.96)***	2.18 (2.17–2.18)***
20–<25 h	1.17 (1.02–1.35)*	4.19 (4.19–4.19)***
25–<30 h	0.99 (0.85–1.16)	1.74 (1.71–1.76)***
30–<35 h	1.05 (0.90–1.23)	1.91 (1.91–1.91)***
35 h or more	Ref.	Ref.
**Employment arrangement at onset of pregnancy**		
Fixed hours	Ref.	Ref.
Flexible hours	1.38 (1.23–1.56)***	1.11 (1.11–1.11)***
**Contract type at the onset of pregnancy**		
Permanent	Ref.	Ref.
Temporary	1.37 (1.25–1.50)***	3.47 (3.41–3.53)***
Unknown	5.21 (0.58–46.97)	27.05 (27.05–27.05)***
**Sick leave during pregnancy**		
Yes	1.57 (1.41–1.75)**	0.00 (0.00–0.00)^b^,***
No	Ref.	Ref.
**Working hours per week one year after childbirth** ^a^		
<12 h	1.34 (1.13–1.59)***	–
12–<20 h	31.64 (27.32–36.63)***	–
20–<25 h	8.78 (7.66–10.08)***	–
25–<30 h	2.49 (2.17–2.86)***	–
30–<35 h	1.23 (1.04–1.45)*	–
35 h or more	Ref.	–
**Employment arrangement one year after childbirth** ^a^		
Fixed hours	Ref.	–
Flexible hours	1.40 (1.27–1.55)***	–
**Contract type one year after childbirth** ^a^		
Permanent	Ref.	–
Temporary	0.96 (0.87–1.07)	–
Unknown	0.57 (0.33–1.00)*	–
**(Additional) Unemployment benefits one year after childbirth**		
Yes	2.60 (2.21–3.06)***	0.02 (0.02–0.02)***
No	Ref.	Ref.
**(Additional) Social assistance benefits one year after childbirth**		
Yes	6.03 (3.62–10.04)***	19001.58 (18934.55–
No	Ref.	19068.84)*** Ref.

Statistical significance is indicated by the following symbols: ***p <0.001, **p <0.01, *p< 0.05.

The model’s area under the curve values were 0.98 and 0.97. The model explained 90% and 81% of the variance in economic independence, as indicated by the Nagelkerke *R*² and McFadden *R*², respectively. Model performance metrics showed high accuracy (0.95), precision (0.96 for economic independence, 0.81 for employment without economic independence, 0.99 for exiting the workforce) and sensitivity (0.98, 0.64 and 0.99 for each respective category).

a For women who exited the workforce, effects of employment characteristics at one year after childbirth could not be directly assessed. This was managed by implementing an interaction effect of employment status (employed versus not employed), allowing these differences to be accounted for in the analyses.

b ORs of 0.00 reflect a boundary estimate under maximum likelihood (for instance, owing to separation or perfect prediction). Interpret with caution.

OR: odds ratio; CI: confidence interval; SES: socioeconomic status; SGA: small for gestational age; Ref.: reference

#### Being employed without economic independence

Women who took sick leave during pregnancy were more likely to be employed without economic independence (odds ratio (OR) = 1.57). Compared with women with high educational attainment, those with low and middle levels of education had much higher odds of falling into this category (ORs of 12.40 and 1.54, respectively). First-generation migrants had significantly higher odds of being employed without economic independence (OR = 1.54) compared with women without a migration background. Work-related characteristics, particularly working fewer hours, were consistently associated with greater economic vulnerability, both at the onset of pregnancy and one year after childbirth. Flexible work arrangements and temporary contracts at the onset of pregnancy also showed this pattern, with flexible arrangements remaining relevant one year later. Regarding pregnancy outcomes, SGA was associated with higher odds of being employed without economic independence (OR = 1.17). Preterm birth was also associated with higher odds of being employed without economic independence (OR = 1.18). Women without a cohabiting partner had higher odds of falling into this category, both at pregnancy onset (OR = 1.35) and one year after birth (OR = 1.22). Other factors, such as younger age, multiparity, lower neighbourhood SES, and receiving additional unemployment or social assistance benefits, were also associated with higher odds of being employed without economic independence, although these associations were generally weaker.

#### Exiting the workforce

Women with low and middle educational attainment had lower odds of exiting the workforce compared with women with high educational attainment (ORs = 0.06 and 0.46 respectively). In contrast, women with a first-generation migration background and those with a second-generation migration background had higher odds of exiting the workforce compared with women who did not have a migration background (ORs = 42.51 and 3.84, respectively). Multiparous women also had higher odds of leaving the workforce (OR = 4.58). Women without a cohabiting partner, either at pregnancy onset (OR = 0.04) or one year postpartum (OR = 0.02), were substantially less likely to exit the workforce. Pregnancy-related outcomes showed mixed associations. Preterm birth was strongly associated with workforce exit (OR = 4.71), while SGA was not related. Work-related characteristics at onset of pregnancy were consistently important. Women working fewer hours, those with flexible arrangements (OR = 1.11) and those with temporary contracts (OR = 3.47) had greater risks of workforce exit. Taking sick leave during pregnancy was not associated with workforce exit, in contrast to remaining employed but without economic independence.

## Discussion

### Interpretation of the findings

The findings in our study highlight a predominant trend that women reduce their working hours after childbirth, especially in the first year. This reduction was apparent independent of the number of hours worked at the onset of pregnancy. Only 11.3% maintained full-time work one year after giving birth, compared with 30.8% at pregnancy onset. Overall, 12.2% exited the workforce entirely. Notably, the reduction in working hours or workforce exit was more pronounced among women with sick leave during pregnancy compared with those without.

Our findings align with prior studies from the UK, US and the Netherlands on the trend of working hour reduction after childbirth [10,24,25]. Our novel contribution lies in the detailed analysis of employment patterns, quantifying the frequency and degree of working hour reductions. Importantly, our analyses highlight that sick leave during pregnancy has long-term consequences for women’s labour market participation. Sick leave during pregnancy was associated with being employed without economic independence one year after childbirth, but not with exiting the workforce. This finding might suggest that women generally remain employed, yet often in more economically vulnerable positions after experiencing sick leave. One possible explanation is that it is important for these women to remain employed to some extent, because this is necessary financially or socially. Health issues during pregnancy may result in reduced working hours, temporary or less secure contracts, or slower career progression. All of these are associated with limited income growth, thus undermining women’s long-term economic independence [26,27].

Our findings show that economic vulnerability after childbirth is not shaped solely by work characteristics, but also by structural and demographic factors. Women with lower educational attainment, a migration background or without a cohabiting partner had higher odds of being employed without economic independence. Interestingly, lower education attainment and not cohabiting with a partner were associated with lower odds of exiting the workforce entirely. This finding suggests that there might be a financial necessity to remain employed even when employment does not ensure economic independence. This finding likely reflects broader societal norms in the Netherlands, where women often serve as primary caregivers and part-time work is common and institutionally supported [28,29]. The Netherlands has had the highest rate (up to 75%) of women working part-time within the European Union in the last decade [30]. This presents a striking contrast with the European average, where merely 28% of employed women aged 15–64 years opt for part-time work [31]. These unique labour patterns underscore the need for country-specific analyses when interpreting work-family balance outcomes.

It is imperative to consider the individual factors affecting women’s economic independence within the broader socio-economic context, such as family dynamics, workplace accommodations and national public policies. The dynamics within families, such as shared parenting responsibilities and broader household support, can both support or hinder women’s effort to balance professional and domestic responsibilities [32]. This balance is also influenced by workplace accommodations, such as flexible work schedules and the option for remote work [25]. Last, public policies, including the duration and pay of parental leave and the accessibility of child care facilities, play pivotal roles in shaping the opportunities of women in the workforce [33]. Understanding the interplay between individual characteristics and broader socio-economic factors is vital for a nuanced interpretation of women’s economic status after childbirth, and for the formulation of policies aimed at supporting women effectively.

The multivariate model revealed some extreme odds ratios, including very high values and ORs of 0.00, which indicate potential limitations or complexities in the data. These unusual outcomes may reflect sparsity, strong underlying barriers, or constraints faced by certain groups, such as first-generation migrants, that the model does not fully capture. Therefore, caution is needed when interpreting these results and further research is recommended to explore these extreme findings in more depth. Furthermore, pregnancy outcomes, such as SGA and preterm birth, showed varying associations with the economic outcomes, suggesting inconsistent impacts that warrant further investigation to interpret these findings.

### Strengths and limitations

A major strength of our study is the large sample size of over 110,000 women in combination with the use of registry data. This provides an objective and reliable depiction of women’s detailed work trajectories after birth and thereby adds novel insights to the literature about workforce participation. Our rich dataset facilitated the analysis of many factors affecting economic independence after childbirth, including demographics, work-related factors and birth outcomes. The results enhance the understanding of how pregnancy, childbirth and sick leave have a lasting influence on women’s workforce participation. While the data did not allow for the distinction between pregnancy-related health issues and non-pregnancy-related health issues as reasons for sick leave, it is important to note that the physical and immunological changes experienced during pregnancy may also increase women’s susceptibility to longer sick leave periods for non-pregnancy-related health issues, such as the flu [34]. Conversely, women with poorer mental or physical health before pregnancy might have a higher risk of pregnancy complications, leading to an increased likelihood of taking sick leave during pregnancy [35].

There were also limitations to our analysis approach. While the register-based nature of our data provides a wide coverage of many topics, they were not primarily collected for scientific research purposes. This restricts certain analyses, as we were unable to explore the reasons leading to the observed changes in working hours or leaving the workforce [36]. Insights into these reasons can form the basis for preventive actions to reduce the proportion of women forced to leave the workforce after childbirth. Furthermore, the registries used in this study did not have information on the mode of delivery, which may impact the woman’s physical recovery time after birth. This potentially affects the ability to resume work and thereby influence the observed patterns in employment resumption and economic independence [36]. However, the most important recovery generally occurs within the first 6–8 weeks after childbirth, regardless of the mode of delivery, and this period typically falls within the legal maternity leave [37]. Additionally, to depict employment patterns spanning several years, we intentionally limited our analysis to data up until 2019 (i.e. pre-COVID). While we aimed to include more recent data, the COVID-19 pandemic significantly disrupted economies worldwide, leading to data gaps and altered work patterns. Early studies show that women took on greater household responsibilities, exacerbating an already uneven domestic labour distribution [16,17]. Another limitation is the restricted meaningfulness of statistically significant results because of our large sample size. Although the large sample enhances statistical power, it also increases the likelihood that small effects reach statistical significance, and it remains unclear whether predictors with low explanatory power would also be statistically significant in a smaller sample. Last, we restricted the study sample to women with paid employment for whom we had monthly income data at the onset of pregnancy. Consequently, our insights do not extend to women who may have started working after childbirth, women with other sources of income or those reliant on benefits – an important demographic for grasping the nuances of economic self-sufficiency as a whole.

### Research and future implications

Our study highlights a critical area for further exploration: the long-term impact of (pregnancy-related) sick leave on women’s re-entry into the workforce. Our findings regarding the influence of sick leave during pregnancy on women’s economic (in)dependence underscore the importance of preventing health issues during pregnancy. In part, preventing (work-related) health issues during pregnancy can be achieved through safer working conditions and accurate workplace accommodations. Research shows that a substantial proportion of women are exposed to physically demanding or unsafe work environments during pregnancy [38]. Preventive measures, including risk assessments early in pregnancy, adjustments in workload and tasks, and offering flexible or remote work arrangements, could reduce the need for prolonged sick leave [39,40]. Strengthening these preventive strategies could therefore play a crucial role in protecting women’s long-term economic independence after childbirth. Furthermore, future studies could aim to disentangle the complex interplay between individual choices, workplace and family policies, and societal norms that affect women’s employment decisions during this critical period. Especially, when external factors hinder the implementation of the preferred work–family balance. Qualitative research could provide more insight into these decisions, how it was experienced and which challenges were most influential.

## Conclusions

Pregnancy and childbirth have a major impact on workforce participation. We observed a marked decrease in working hours, particularly in the initial year after childbirth. This trend is especially pronounced among those with sick leave during pregnancy. Further investigation is necessary to comprehend these findings fully and eventually enable the development of more effective workplace and family policies, for example, flexible work arrangements or prolonged partner leave, that support women in maintaining their role in the workforce. Understanding the interplay between individual characteristics and broader socioeconomic factors is pivotal for the formulation of such policies.

## Supplemental Material

sj-docx-1-sjp-10.1177_14034948251394645 – Supplemental material for Patterns of women’s work resumption and economic independence after childbirth: A longitudinal study Supplemental material, sj-docx-1-sjp-10.1177_14034948251394645 for Patterns of women’s work resumption and economic independence after childbirth: A longitudinal study by Lindsey Van Der Meer, Kajal S.C. Mohabier, Adja J.M. Waelput, Alex Burdorf, Eric A.P. Steegers and Loes C.M. Bertens in Scandinavian Journal of Public Health
