# Supplementary material for: Patterns of women’s work resumption and economic independence after childbirth: A longitudinal study
Source: Scand J Public Health. 2025 Dec 2;54(8):832–44. doi: 10.1177/14034948251394645 (PMC13710720; doi:10.1177/14034948251394645)
Supplement: Supplementary file 1 [file sj-docx-1-sjp-10.1177_14034948251394645.docx]

**SUPPLEMENTARY FILES**

| **Box 1.** Policies and definitions | |
| --- | --- |
| Economic Independence | In the Netherlands, a person is considered to be economically independent if the **individual** net income level from labour and from business activities is at least the same as according to policy values applied to individual minimum income or higher. This threshold value is set as the equivalent of 70% of legal minimum wage, i.e. the net amount of social assistance provided to a single person.^1^ Economic independence not only provides autonomy and a sense of security, it also contributes to improved health and well-being of individuals on both the short and long term.^2^  *Note: While the number of children can influence a woman's economic independence, factors such as employment opportunities, childcare support, access to education and training, and social support networks also play significant roles in shaping women's economic outcomes.* |
| Maternity leave | Women in paid employment, women on social security, and self-employed women (i.e. business owners), are entitled to 16 weeks of maternity leave of which at least 4 weeks before the estimated due date and 10 weeks after childbirth, in case of a singleton pregnancy. For women expecting multiple births, the period of maternity leave is extended to 20 weeks, with at least 8 weeks before the estimated due date and 10 weeks after childbirth.^3^  Women in paid employment are entitled to full income compensation during maternity leave. Women on social security receive 100% of their social security benefits, and self-employed women are entitled to the subsistence minimum wage or a percentage thereof, depending on their work history. |
| Partner leave | Partners in paid employment are entitled to one week of fully paid leave, which can be extended to up to five additional weeks at 70% of the salary. Self-employed partners do not have this right and are responsible for arranging their own leave and income.^3^ |
| Parental leave | Parents are also entitled to 26 weeks of parental leave during the first eight years of their child's life. Of this period, nine weeks are partially paid at up to 70% of the salary if they are taken within the first year of the child's life. For self-employed parents, these rights may differ depending on their work history and insurance arrangements.^3^ |
| Childcare allowance | Parents in paid employment or self-employed parents are entitled to a childcare allowance to cover part of the subsidized childcare costs.^4^ |
| Sickness benefit | In case of sick leave during pregnancy, women in paid employment can seek financial assistance from the Dutch government in the form of sickness benefit. The sickness benefit amounts to 100% of the regular salary, with a maximum duration of 2 years. Sick leave during pregnancy includes pregnancy-related health issues as well as non-pregnancy-related health issues.^5^ |
| Unemployment benefit | In case of involuntary loss of employment, women can seek financial assistance from the Dutch government in the form of unemployment benefit. The unemployment benefit amounts to 75% of the regular salary for 2 months and 70% thereafter. This benefit is provided for a minimum of 3 months up to 24 months based on the women’s employment history, with the prerequisite that the women actively seek work and pursue economic independence.^6^  *Note: If the earnings of a new job are equal to or lower than 87.5% of the monthly unemployment benefit, the income will be supplemented by a partial unemployment benefit (i.e. additional unemployment benefit).* |
| Social assistance benefit | The social assistance benefit amounts 70% of the net minimum wage and is applicable for women who do not have enough income to provide for their basic needs.^7^  *Note: Individuals with income from work might also apply for an additional social assistance benefit if their income is lower than the statutory subsistence minimum (i.e. additional social assistance benefit).* |
| Statutory subsistence minimum | For the years 2015 to 2019 the statutory subsistence minimum was defined as a monthly earning of €920, €940, €950, €960, and €980 or more, respectively for each year. |

| Supplementary File 2. Work related characteristics of women in paid employment (N = 114,722) up to three years after childbirth | | | | |
| --- | --- | --- | --- | --- |
| Characteristics | | **One year after childbirth** | **Two years after childbirth** | **Three years after childbirth** |
| Working hours per week, n (%) | |  |  |  |
| < 12 hours | | 5,552 (4.8%) | 5,216 (4.6%) | 5,133 (4.5%) |
| 12 - < 20 hours | | 13,371 (11.7%) | 12,803 (11.2%) | 12,987 (11.3%) |
| 20 - < 25 hours | | 31,529 (27.5%) | 32,432 (28.3%) | 32,811 (28.6%) |
| 25 - < 30 hours | | 19,040 (16.6%) | 18,798 (16.4%) | 18,585 (16.2%) |
| 30 - < 35 hours | | 18,279 (15.9%) | 18,128 (15.8%) | 17,345 (15.1%) |
| 35 hours or more | | 12,968 (11.3%) | 12,753 (11.1%) | 11,791 (10.3%) |
| Not employed | | 13,983 (12.2%) | 14,592 (12.7%) | 16,070 (14.0%) |
| Employment arrangement, n (%) | |  |  |  |
| Fixed hours | | 72,927 (63.6%) | 71,623 (62.4%) | 72,024 (62.8%) |
| Flexible hours | | 27,812 (24.2%) | 28,507 (24.9%) | 26,628 (23.2%) |
| Not employed | | 13,983 (12.2%) | 14,592 (12.7%) | 16,070 (14.0%) |
| Contract type, n (%) | |  |  |  |
| Permanent | | 74,825 (65.2%) | 72,933 (63.6%) | 73,322 (63.9%) |
| Temporary | | 25,730 (22.4%) | 26,919 (23.5%) | 24,948 (21.8%) |
| Unknown | | 184 (0.2%) | 278 (0.2%) | 382 (0.3%) |
| Not employed | | 13,983 (12.2%) | 14,592 (12.7%) | 16,070 (14.0%) |
| Sick leave, n (%) | |  |  |  |
| Yes | | 676 (0.6%) | 821 (0.7%) | 663 (0.6%) |
| (Additional) Unemployment benefits, n(%) | | | | |
| Yes | | 5,941 (5.2%) | 3,522 (3.1%) | 3,226 (2.8%) |
| (Additional) Social assistance benefits, n(%) | | | | |
| Yes | | 1,160 (1.0%) | 1,184 (1.0%) | 1,197 (1.0%) |
| Cohabiting partner, n (%) | |  |  |  |
| Yes | | 106,921 (93.2%) | 105,305 (91.8%) | 98,749 (86.1%) |
| Working hours partner^1^, n (%) | |  |  |  |
| < 12 hours | | 886 (0.8%) | 783 (0.7%) | 661 (0.7%) |
| 12 -< 20 hours | | 1,083 (1.0%) | 972 (0.9%) | 871 (0.9%) |
| 20 -< 25 hours | | 1,713 (1.6%) | 1,657 (1.6%) | 1,539 (1.6%) |
| 25 -< 30 hours | | 2,734 (2.6%) | 2,742 (2.6%) | 2,625 (2.7%) |
| 30 -< 35 hours | | 11,715 (11.0%) | 11,641 (11.1%) | 11,316 (11.5%) |
| 35+ hours | | 72,114 (67.5%) | 70,683 (67.1%) | 65,416 (66.2%) |
| Not employed | | 16,676 (15.6%) | 16,827 (16.0%) | 16,321 (16.5%) |
| Economic independence, n (%) | |  |  |  |
|  | Yes | 89,987 (78.4%) | 90,361 (78.8%) | 89,513 (78.0%) |
|  | No | 10,752 (9,4%) | 9,769 (8,5%) | 9,139 (8.0%) |
|  | Not employed | 13,938 (12.2%) | 14,592 (12.7%) | 16,070 (14.0%) |
| ^1.^ These figures represent only the weekly working hours of partners of women who were cohabiting. | | | | |

| **Supplementary File 3.** Univariate and multivariate multinomial logistic regression of economic independence one year after childbirth (N = 113,134). | | | | |
| --- | --- | --- | --- | --- |
| Variables | Univariate multinomial logistic regression | | Multivariate multinomial logistic regression | |
|  | | | | |
|  | **Employed without economic independence**  **OR (95% CI)** | **Exited the workforce**  **OR (95% CI)** | **Employed without economic independence**  **OR (95% CI)** | **Exited the workforce**  **OR (95% CI)** |
| Individual characteristics | | | | |
| **Age** | | | | |
|  | 0.88 (0.88-0.89) *** | 0.92 (0.92-0.92) *** | 0.92 (0.91-0.93) *** | 1.01 (0.51-1.97) |
| **Generational migration status** | | | | |
| Dutch  First-generation migrant  Second-generation migrant | Ref  1.51 (1.40-1.63)  ***  1.13 (1.04-1.22)  *** | Ref  3.74 (3.53-3.96)  ***  2.32 (2.18-2.47)  *** | Ref  1.54 (1.35-1.77) ***  1.09 (0.95-1.24) | Ref  42.51 (42.36-42.66)  ***  3.84 (3.83-3.84) *** |
| **Educational attainment** | | | | |
| Low  Middle  High  Unknown | 14.96 (13.69-16.35)  ***  5.84 (5.47-6.24)  ***  Ref  3.52 (3.19, 3.89)  *** | 7.49 (6.95-8.07)  ***  2.44 (2.32-2.57) ***  Ref  1.59 (1.45-1.73)  *** | 12.40 (10.77-14.29)  ***  4.08 (3.70-4.49) ***  Ref  5.23 (4.60-6.07) *** | 0.06 (0.06-0.06)  ***  0.46 (0.45-0.46) ***  Ref  0.08 (0.08-0.08) *** |
| **Cohabiting partner at onset of pregnancy** | | | | |
| Yes  No | Ref  2.06 (1.93-2.20) *** | Ref  2.95 (2.79-3.12) *** | Ref  1.35 (1.18-1.55) *** | Ref  0.04 (0.04-0.04) *** |
| **Parity** | | | | |
| Primiparous  Multiparous | Ref  1.16 (1.11-1.22) *** | Ref  0.83 (0.79-0.87) *** | Ref  0.83 (0.76-0.91) *** | Ref  4.58 (4.51-4.64) *** |
| **Neighbourhood SES** | | | | |
|  | 0.62 (0.55-0.69) *** | 0.27 (0.25-0.30)  *** | 0.75 (0.62-0.91) *** | 0.00 (0.00-0.00) **^†^** *** |
| **Perinatal mortality** | | | | |
| Yes  No | 1.40 (0.94-2.10)  Ref | 1.25 (0.85-1.84) Ref | - | - |
| **SGA** | | | | |
| Yes  No | 1.23 (1.12-1.34) *** Ref | 1.36 (1.26-1.47) ***  Ref | 1.17 (1.02-1.35)  *  Ref | 0.00 (0.00-0.00) **^†^** ***  Ref |
| **Preterm birth** | | | | |
| Yes  No | 1.13 (1.02-1.26)  *  Ref | 1.35 (1.23-1.48) ***  Ref | 1.18 (1.00-1.40)  *  Ref | 4.71 (4.71-4.71) ***  Ref |
| **Cohabiting partner one year after childbirth** | | | | |
| Yes  No | Ref  2.22 (2.03-2.41)  *** | Ref  3.70 (3.45-3.96)  *** | Ref  1.22 (1.02-1.45)  * | Ref  0.02 (0.02-0.02)  *** |
| Work-related characteristics | | | | |
| **Working hours per week at the onset of pregnancy** | | | | |
| <12 hours  12 - < 20 hours  20 - < 25 hours  25 - < 30 hours  30 - < 35 hours  35 hours or more | 55.17 (49.62-61.34)  ***  15.33 (14.00-16.79)  ***  3.24 (2.95-3.56) ***  2.02 (1.81-2.25) ***  1.36 (1.22-1.52)  ***  Ref | 15.82 (14.49-17.28)  ***  3.34 (3.15-3.64) ***  1.19 (1.11-1.28) ***  0.90 (0.85-0.98) ***  0.91 (0.85-0.98) ***  Ref | 2.71 (2.30-32.0) ***  1.70 (1.48-1.96) ***  1.17 (1.02-1.35)  *  0.99 (0.85-1.16)  1.05 (0.90-1.23)  Ref | 22.63 (22.55-22.70)  ***  2.18 (2.17-2.18) ***  4.19 (4.19-4.19) ***  1.74 (1.71-1.76) ***  1.91 (1.91-1.91) ***  Ref |
| **Employment arrangement at onset of pregnancy** | | | | |
| Fixed hours  Flexible hours | Ref  4.67 (4.38-4.98) *** | Ref  5.44 (5.13-5.76) *** | Ref  1.38 (1.23-1.56) *** | Ref  1.11 (1.11-1.11) *** |
| **Contract type at the onset of pregnancy** | | | | |
| Permanent  Temporary  Unknown | Ref  2.74 (2.61-2.87) ***  10.98 (0.23-4.12) | Ref  4.42 (4.22-4.63) ***  0.01 (0.00-3711.08) | Ref  1.37 (1.25-1.50)  ***  5.21 (0.58-46.97) | Ref  3.47 (3.41-3.53) ***  27.05 (27.05-27.05)  *** |
| **Sick leave during pregnancy** | | | | |
| Yes  No | 4.47 (4.19-4.76) *** Ref | 7.71 (7.30-8.15) ***  Ref | 1.57 (1.41-1.75)  **  Ref | 0.00 (0.00-0.00) **^†^** ***  Ref |
| **Working hours per week one year after childbirth^1^** | | | | |
| <12 hours  12 - < 20 hours  20 - < 25 hours  25 - < 30 hours  30 - < 35 hours  35 hours or more | 1.54 (1.31-1.82) ***  38.88 (33.89-44.62)  ***  10.42 (9.14-11.87) ***  2.79 (2.44-3.19) ***  1.34 (1.14-1.57) ***  Ref | -  -  -  -  -  - | 1.34 (1.13-1.59) ***  31.64 (27.32-36.63)  ***  8.78 (7.66-10.08) ***  2.49 (2.17-2.86) ***  1.23 (1.04-1.45)  *  Ref | -  -  -  -  -  - |
| **Employment arrangement one year after childbirth^1^** | | | | |
| Fixed hours  Flexible hours | Ref  1.94 (1.89-1.99) *** | -  - | Ref  1.40 (1.27-1.55) *** | -  - |
| **Contract type one year after childbirth^1^** | | | | |
| Permanent  Temporary  Unknown | Ref  1.76 (1.71-1.80) ***  0.92 (0.65-1.32) | -  -  - | Ref  0.96 (0.87-1.07)  0.57 (0.33-1.00)  * | -  -  -  - |
| **(Additional) Unemployment benefits one year after childbirth** | | | | |
| Yes  No | 4.71 (4.26-5.19) ***  Ref | 15.56 (15.38-17.83)  *** Ref | 2.60 (2.21-3.06) ***  Ref | 0.02 (0.02-0.02) ***  Ref |
| **(Additional) Social assistance benefits one year after childbirth** | | | | |
| Yes  No | 0.02 (0.01, 0.03) ***  Ref | 2.42 (2.04, 2.86) ***  Ref | 6.03 (3.62-10.04) ***  Ref | 19001.58 (18934.55-19068.84)  ***  Ref |
| **Ref = Reference.**  **Statistical significance is indicated by the following symbols: *** p < 0.001, ** p < 0.01, * p < 0.05.**  **The model's Area Under the Curve (AUC) values were 0.98 and 0.97. The model explained 90%, 81%, and 66% of the variance in economic independence, as indicated by the Nagelkerke R², McFadden R², and Maximum Likelihood R², respectively. Model performance metrics showed high accuracy (0.95), precision (0.96 for economic independence, 0.81 for employment without economic independence, 0.99 for exiting the workforce), and sensitivity (0.98, 0.64, and 0.99 for each respective category).**  **^1.^ For women who exited the workforce, effects of employment characteristics at one year after childbirth could not be directly assessed. This was managed by implementing an interaction effect of employment status (employed vs. not employed), allowing these differences to be accounted for in the analyses.**  **^†^ORs of 0.00 reflect a boundary estimate under maximum likelihood (for instance, due to separation or perfect prediction). Interpret with caution.** | | | | |

**References**

1. Centraal Bureau voor de Statistiek (CBS). Economically independent. 2018 [Available from: <https://www.cbs.nl/en-gb/our-services/methods/definitions/economically-independent> ].
2. Irshad Ahmad R, Dr TS. Economic empowerment of woman: A review of current research. International Journal of Educational Review, Law And Social Sciences (IJERLAS). 2023;3(2):601-5.
3. UWV (Employee Insurance Agency). **Maternity and parental leave.** UWV. [Available from: <https://www.uwv.nl/en/individuals/maternity-and-parental-leave>]
4. Dienst Toeslagen (Belastingdienst). **My child goes to a childcare centre**. [Available from: [https://www.belastingdienst.nl/wps/wcm/connect/bldcontenten/belastingdienst/individuals/benefits/moving_to_the_netherlands/my_child_goes_to_a_childcare_centre/](https://www.belastingdienst.nl/wps/wcm/connect/bldcontenten/belastingdienst/individuals/benefits/moving_to_the_netherlands/my_child_goes_to_a_childcare_centre/?utm_source=chatgpt.com)]
5. UWV (Employee Insurance Agency). **Sickness benefit.** UWV. [Available from: <https://www.uwv.nl/en/individuals/sickness-benefit>]
6. UWV (Employee Insurance Agency)**. Unemployment benefit.** UWV. [Available from: <https://www.uwv.nl/en/individuals/unemployment-benefit>]
7. Rijksoverheid. Wanneer heb ik recht op bijstand?. [Available from: <https://www.rijksoverheid.nl/onderwerpen/bijstand/vraag-en-antwoord/wanneer-heb-ik-recht-op-bijstand>]
